# Suicidal Ideation in Dementia Family Caregivers

**DOI:** 10.1093/geroni/igaa057.1545

**Published:** 2020-12-16

**Authors:** Frank Puga, Danny Wang, Carolyn Pickering

**Affiliations:** 1 The University of Alabama at Birmingham, Birmingham, Alabama, United States; 2 University of Alabama at Birmingham, Birmingham, Alabama, United States

## Abstract

Family caregivers of individuals living with Alzheimer’s disease or related dementias (ADRDs) are exposed to unique stressors that put them at risk for depression and suicidal ideation. To date, little is known about contextual factors surrounding suicidal ideation among ADRD family caregivers. We investigated individual caregiver characteristics (gender, age, relationship to care-recipient, history of depression and anxiety) and daily environmental stressors (behavioral symptoms of dementias; BSDs) associated with daily suicidal ideation using a micro-longitudinal design and ecological momentary assessment methods. Data were collected from a national sample of family caregivers (N=51) who completed daily diaries over 21 days (n=911). Suicidal ideation was endorsed on forty-seven days (5.16%) during the sampling period, with 11 participants (22%) endorsing suicidal ideation at least once. Suicidal ideation did not differ based on the caregiver’s age and relationship to the care-recipient (spouse or child). Participants with a history of mild depression and anxiety endorsed more days with suicidal ideation. Finally, family caregivers were more likely to endorse suicidal ideation on a day when more than one type of BSD was reported (OR = 1.25, 95% CI: 1.04-1.50, p = 0.018) and when BSDs were perceived as more bothersome than average (OR = 1.12, 95% CI: 1.05-1.19, p < 0.001). In this investigation, we identified descriptive and predictive factors that will inform the development of targeted interventions for ADRD caregivers at high risk of suicidal ideation.

